# Dextrose Prolotherapy for Symptomatic Grade IV Knee Osteoarthritis: A Pilot Study of Early and Longer-Term Analgesia and Pain-Specific Cytokine Concentrations

**DOI:** 10.3390/clinpract12060097

**Published:** 2022-11-14

**Authors:** Gastón Andrés Topol, Ines Guerrero Pestalardo, Kenneth Dean Reeves, Fernando Elias, Neven J. Steinmetz, An-Lin Cheng, David Rabago

**Affiliations:** 1 Department of Physical Medicine and Rehabilitation, National University of Rosario, Rosario CP2000, Argentina; 2 Private Practice of Physical Medicine and Rehabilitation, Rosario C2000, Argentina; 3 Private Practice of Physical Medicine and Rehabilitation, Roeland Park, KS 66205, USA; 4 Laboratorio CIBIC S.A., Rosario C2000, Argentina; 5 Regenexx, Broomfield, CO 80021, USA; 6 Department of Biomedical and Health Informatics, University of Missouri-Kansas City, Kansas City, MO 64108, USA; 7 Department of Family and Community Medicine, Penn State College of Medicine, Hershey, PA 17003, USA

**Keywords:** glucose, neurogenic inflammation, neuropeptide Y, cytokine, nerve

## Abstract

Background: Neurocytokines may upregulate or downregulate neuropathic pain. We hypothesized that dextrose (D-glucose) injections for therapeutic purposes (dextrose prolotherapy: DPT) in painful knee osteoarthritis (KOA) would favorably affect synovial-fluid neurocytokine concentrations. Methods: Twenty participants with grade IV symptomatic KOA received synovial-fluid aspiration followed by dextrose or simulated dextrose injections, followed by the reverse after one week. All participants then received open-label dextrose injections monthly for 6 months, with serial assessments of walking pain at 20 min for 9 months, as well as synovial-neurocytokine-concentration measurements (calcitonin gene-related peptide, substance P (SP), and neuropeptide Y (NPY)) at one week and three months. Results: Clinically important analgesia was observed at 20 min and for 9 months post dextrose injection. One -week synovial-fluid SP concentration rose by 111% (*p* = 0.028 within groups and *p* = 0.07 between groups) in the dextrose-injected knees compared to synovial-fluid aspiration only. Three-month synovial-fluid NPY concentration dropped substantially (65%; *p* < 0.001) after open-label dextrose injection in all knees. Conclusions: Prompt and medium-term analgesia after intra-articular dextrose injection in KOA was accompanied by potentially favorable changes in synovial-fluid neurocytokines SP and NPY, respectively, although these changes were isolated. Including neurocytokines in future assessments of DPT to elucidate mechanisms of action is recommended.

## 1. Introduction

Knee osteoarthritis (KOA) affects around 30% of adults by age 65 [1] and is responsible for substantial chronic pain and disability worldwide [2]. Identifying conservative and safe care that complements current strategies is a top priority in research [3]. Dextrose prolotherapy (DPT), the injection of hypertonic dextrose for therapeutic purposes, has outperformed anesthetic [4], steroid [5], or saline injections [6,7], and exercise controls [8,9] in multiple randomized trials for improvements in both function and pain in knee osteoarthritis. An important and frequent empirical observation is the prompt analgesic effect after dextrose injection in a variety of pain conditions, including patients with painful osteoarthritic knees. Why dextrose injections would reduce pain in KOA is unknown. 

The injection of 5 percent dextrose in water (D5W) into the caudal epidural space has been reported to have an analgesic effect within 15 min in patients with low back and buttock or leg pain in masked comparison with normal saline [10]. A similar analgesic effect was reported within 5 min after hydrodissection with D5W of the stellate ganglion, brachial plexus, cervical nerve roots, and paravertebral spaces in patients with neurogenic pain [11]. 

Dr. John Lyftogt was the first to suggest an immediate (within seconds) neurogenic effect of dextrose injections in a series of publications describing the treatment of consecutive patients with chronic shoulder, elbow, low back, knee, and Achilles pain with a subcutaneous injection of dextrose into painful superficial-sensory nerves [12,13,14]. He proposed that dextrose injections affect chronic pain by altering the concentration of inflammatory or anti-inflammatory neurocytokines [14,15], which are small proteins produced and released by nociceptive sensory afferent nerves [16]. Such a mechanism may also explain the prompt analgesia empirically observed after hypertonic dextrose injection in painful KOA. Although randomized trials report the benefit of the intra-articular and periarticular injection of dextrose in the medium- and long-term reduction of KOA pain [4,5,6,7,8,9], the speed of the onset of analgesia has never been assessed, and the mechanism of prompt and medium-term analgesia is unclear. Specifically, the measurement of changes in anabolic and catabolic cytokines (complex polypeptides that powerfully affect cellular function) in response to intra-articular injections of dextrose in KOA has been reported only once in humans, in a non-controlled report of cytokine changes in seven knees [17]. 

Changes in multiple neurocytokine concentrations are associated with KOA. Substance P (SP) concentration is elevated in the synovial fluid in osteoarthritic knees [18], calcitonin gene-related peptide (CGRP) concentration increases in both the infrapatellar fat pad and synovial cells as the Kellgren–Lawrence grade increases [19], and the synovial-fluid concentration of neuropeptide Y (NPY) increases as pain severity increases in osteoarthritic knees [20]. 

In this study, we treated patients with severe KOA using a two-phase protocol. In the early phase (0–1 week), we used a randomized, controlled methodology comparing Dextrose-1st participants to Aspiration-1st participants (with simulated dextrose injections), and in the second phase (1 week to 9 months), we employed an open-label protocol in which all participants received dextrose injections. We hypothesized that the Dextrose-1st participants would experience prompt (20 min) and significant analgesia in a controlled comparison with the Aspiration-1st participants, that both groups would report substantial analgesia over 9 months (associated with the periodic open-label injection of dextrose over 6 months), and that significant and favorable changes in synovial-fluid neurocytokine measures (SP, CGRP, and NPY) would be the result at 1 week and 3 months. 

## 2. Materials and Methods

### 2.1. Inclusion Criteria

Inclusion criteria included adults 50 to 80 years of age with 6 or more months of knee pain with walking (knee pain ≥ 6 on a 0–10-point numerical rating scale (NRS)), a weight-bearing radiograph consistent with high-grade medial-compartment cartilage loss (Kellgren–Lawrence grade 4), the confirmation of exposed subchondral bone by ultrasound at 110 degrees of flexion, and an easily visible suprapatellar pouch with quadriceps contraction, using a roll behind the knee for popliteal compression. Exclusion criteria included a current intake of NSAIDs or steroids, current anticoagulation therapy, inflammatory or post-infectious knee arthritis, systemic inflammatory conditions, knee flexion of less than 100 degrees, knee extension of less than 165 degrees, any valgus, varus of more than 15 degrees, any knee injection in the preceding 3 months, BMI over 40 kg/m^2^, gross synovial folds seen on ultrasound, elevation of sedimentation rate, C-reactive protein, rheumatoid factor, or antinuclear antibodies. 

### 2.2. Screening, Group Assignment, Data Gathering, and Group Allocation

Recruitment began on 1 August 2018 from the private practice of the primary investigator/treating physician (GAT) and via referral from the Instituto de Fisiatría y Traumatología in Rosario, Argentina. 

**Figure 1:**
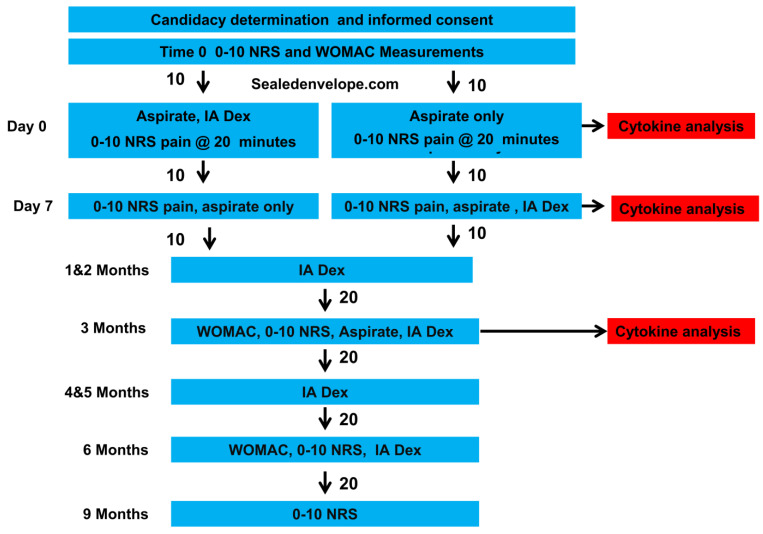
Consort flow chart.

The primary investigator (GAT) determined eligibility after an assessment of patient history, review of plain films, manual examination, and ultrasound examination (Figure 1).

Potential participants were then offered study participation. Interested persons were provided informed consent forms by the research coordinator (IGP). Baseline pain and functional data were gathered by the research coordinator, who subsequently allocated the participants 1:1 in two blocks of 10 using an internet-based random allocator (sealedenvelope.com) to immediate or delayed (by one week) dextrose injections. If knees were treated bilaterally, they received the same treatment, although synovial-fluid cytokine analysis was performed only on the knee that was more symptomatic at baseline due to limitations in the number of wells available within the ELISA kits. Participants, the office manager, and laboratory personnel were masked during the initial group assignment; the injector and outcome assessor were not.

### 2.3. Synovial-Fluid Aspiration and Injection Method

Participants were told, “We will withdraw fluid, and you may or may not be receiving dextrose injection through the same needle”. The injector manipulated syringes at the side of the table at tabletop height, and the ultrasound cart also prevented the participants from viewing procedural details. Participants were asked to look away from their knee during the procedure. After sterile preparation, 5.5 mL of synovial fluid was aspirated from the suprapatellar pouch under ultrasound guidance, with the screen turned away from the patient. The Dextrose-1st participants were immediately injected with 10 mL of 12.5% dextrose without comment, while the Aspiration-1st participants received a simulated injection using an empty 10 mL syringe. The procedural time was similar in each case. At the 1-week follow-up and after pain data were gathered, the initial group allocation was revealed to the participants, 2 mL of synovial fluid was aspirated for analysis, and the injection of 10 mL of 12.5% dextrose was done only in those who received a simulated injection at baseline. At 1, 2, 3, 4, 5, and 6 months, all participants were offered and elected to receive repeat D5W intra-articular injections via the suprapatellar pouch. After injections, patients were advised to use acetaminophen as needed, to avoid NSAIDs, and to minimize forceful and repetitive use of their knees for three days. 

### 2.4. Primary and Secondary Measures

The primary clinical measure for analgesic effects was the NRS for walking pain obtained pre-injection, 20 min post-injection, and at one week, 3, 6, and 9 months, where 0 meant “no pain or dysfunction” and 10 meant “the worst pain or dysfunction imaginable”. The NRS is commonly used to measure treatment-related improvements in musculoskeletal pain, with a 33% improvement associated with “much less pain” [21], and a 0–10 NRS ordinal improvement of 3.3 points representing twice the minimal clinically important difference (MCID) of 1.65 points [22]. The primary cytokine measures were synovial-fluid concentrations of neurocytokines CGRP, SP, and NPY at 0 weeks, 1 week, and 3 months. The secondary clinical measure was the 0–100 Western Ontario and McMaster Universities Arthritis Index (WOMAC), obtained at 0, 3, and 6 months, with an MCID for the composite score after rehabilitation efforts of 12.0 [23]. Secondary cytokine measures included synovial concentrations of transforming growth factor-beta (TGF-β), insulin-like growth factor-1 (IGF-1), tissue inhibitor of metalloproteinase-1 (TIMP-1), matrix metalloproteinase-3 (MMP-3), and interleukin-6 (IL-6) concentrations at 0 weeks, 1 week, and three months. 

### 2.5. ELISA and Total Protein Testing

Synovial-fluid samples were split into 0.5 mL aliquots, placed in a medical freezer until the end of each clinic day, and then carried on dry ice to the CIBIC laboratory (Rosario, Argentina) where they were frozen at −70 degrees centigrade. All synovial-fluid samples were digested prior to ELISA analysis using 10% hyaluronidase (Stem Cell Technologies; Cambridge, MA, USA) per the manufacturer’s specifications. Concentrations of CGRP, NPY (BioSource; Vancouver, BC, Canada) and SP, as well as TGF-β, IGF-1, TIMP-1, MMP-3, and IL-6 (R&D Systems; Minneapolis, MN, USA), were analyzed using the manufacturer’s ELISA protocols. Total protein analysis (Thermo Fisher Scientific Pierce BCA kit; Waltham, MA, USA) was performed to normalize the reporting of the ELISA measurements.

### 2.6. Analysis

Previous data on neurocytokine changes after DPT were not available. Sample size was determined by convenience and resource limitations and was limited to 10 in each group in this pilot data collection. Data were analyzed using PASW Statistics 18, Release 18.0.0, IBM, and SAS version 9.4. Analysis was performed using the intention-to-treat approach. A between-group analysis for differences in baseline characteristics was performed using t-tests for normal data, Mann–Whitney tests for Likert scale data, and Pearson’s chi-squared test for categorical variables. Baseline characteristics that met or approached a significant difference between groups were included as covariates in the follow-up analyses. Mann–Whitney tests were performed for the NRS raw scores to test the difference from the baseline to different time points. For the NRS raw scores, differences between the baseline and each follow-up time point (20 min, one week, and 3, 6 and 9 months) were calculated and treated as outcome variables in the proportional odds model to estimate the significance of between-group differences. Differences between the baseline and each follow-up time point (3 and 6 months for the WOMAC) were calculated and treated as outcome variables in the general linear model analyses. The change scores in cytokine data were not normally distributed. *p*-values for the changes in cytokine concentrations were calculated by Mann–Whitney testing for the between-group comparisons from 0 to 1 week, and by Wilcoxon matched-pair signed-ranks test for the within-group analyses from 0 to 3 months. A Bonferroni-corrected alpha of 0.006 (0.05/8) was utilized for the determination of significance for the within and between-group analysis of change scores for the eight cytokines analyzed. 

## 3. Results

Data analysis was completed on 23 November 2021. Randomization produced similar groups of the middle-to-older-aged participants (68 ± 9 years, 45% male) with an unremarkable BMI (31 ± 3 kg/m^2^), moderate-to-severe pain (8.1 ± 1.4 points), and moderate-to-severely elevated WOMAC scores (54 ± 18 points) (Table 1). The gender distribution was equally balanced in each group (5/10 females in the Dextrose-1st group and 6/10 females in the Aspiration-1st group. The baseline NRS scores tended to be higher in the Aspiration-1st group, approaching clinical significance, and the baseline NRS score was included as a covariate for statistical analysis. Side effects were not reported, although they were not formally followed. 

### 3.1. Analgesic Effects of Dextrose

**Figure 2:**
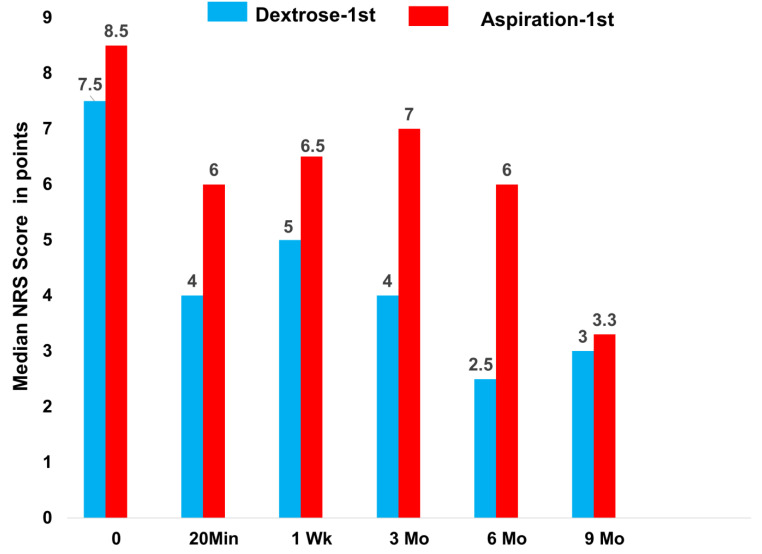
Median NRS pain scores from 0 to 9 months for Dextrose-1st group, given dextrose at time 0 and, then monthly for 6 months, versus Aspiration-1st group, given dextrose at one week and then monthly for 6 months.

Prompt analgesia was measured 20 min post-injection in the Dextrose-1st group, as evidenced by the improvement in NRS score. (Figure 2) The Aspiration-1st group had also improved substantially by 20 min post-injection. The Dextrose-1st group outperformed the Aspiration-1st group for the between-group difference in median raw-score improvement (*p* = 0.05; Table 2) at 20 min. The magnitude of the median NRS score for pain improvement in the Dextrose-1st group by 20 min post-injection (3.5) was clinically meaningful, in contrast with a minimal clinically important difference (MCID) of 1.5 [22]. 

Analgesia in both groups had waned by one week (Figure 2; Table 2). Because both groups had a received dextrose injection by one week, and all participants received monthly dextrose injections after that over 6 months, we expected that improvements in both groups would approximate each other by three months. Although the Dextrose-1st group trended somewhat better over 9 months than the Aspiration-1st group, the between-group differences did not reach significance for an improvement in median NRS pain scores at 3 months (*p* = 0.37), 6 months (*p* = 0.19), or 9 months (*p* = 0.44), and values at 3, 6, and 9 months were combined for the evaluation of median changes. The significance of these improvements is shown in Table 2. Figure 2 and Table 2 indicate that, with continued dextrose injections over 6 months, evidence for dextrose-injection-related analgesia was strong after the 9-month follow-up.

### 3.2. Effects of Dextrose Injection and Aspiration Only on Synovial-Fluid Cytokine Levels by 1 Week

The changes in neurocytokines (SP, CGRP, and NPY) and selected non-neurocytokines (MMP-3, TIMP-1, IL-6, IGF, and TGFβ) from 0 weeks to 1 week are shown in Table 3. SP concentration increased by near significance by one week within the Dextrose-1st group (*p* = 0.028), and the between-group difference also approached significance (*p* = 0.07). No other neurocytokine or non-neurocytokine changes approached both within-group and between-group significance. 

### 3.3. Effect of Dextrose Injection on Cytokine Levels at 3 Months

The Dextrose-1st and Aspiration-1st groups both received three dextrose injections before synovial-fluid sampling at three months and were combined for the analysis of changes in neurocytokines and selected non-neurocytokines from 0–3 months (Table 4). Of the three neurocytokines evaluated, the SP and CGRP changes were not significant, but the concentration of NPY dropped from 7.5 ± 6.6 to 2.6 ± 4.2 picograms; *p* < 0.001. (Table 4). A significant elevation of an anti-inflammatory/anabolic cytokine (IGF; 56%; *p* = 0.003; Table 4) was also observed.

### 3.4. Effect of Dextrose Injection on the WOMAC Score

**Figure 3:**
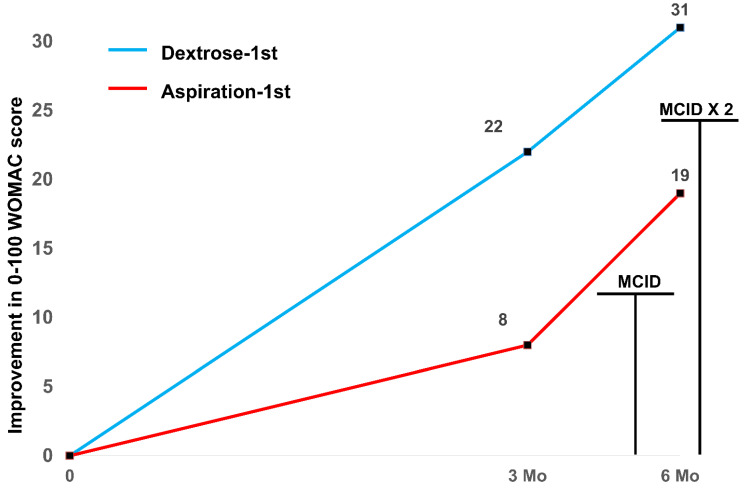
Change in the mean WOMAC score from time 0 through 6 months for combined groups (n = 20) with MCID levels indicated.

Figure 3 depicts the amount of reduction (improvement) in the WOMAC score (numerical data in Table 5). Because both groups received open-label dextrose injections after 1 week, we anticipated no significant between-group difference would be found at three or six months. Although a significant between-group difference for the improvement in the WOMAC score by 3 months favoring the Dextrose-1st group (22 ± 6 vs. 8 ± 4; *p* = 0.03) was found, there was no longer a significant between-group difference by 6 months (31 ± 4 vs. 18 ± 4; *p* = 0.09). Upon combining the two groups, the 6-month mean improvement in the WOMAC was 25 ± 3.1 points, a change that exceeded twice the MCID for WOMAC-assessed improvement.

## 4. Discussion

The randomized, controlled, and blinded portion of this study confirmed a prompt and clinically meaningful improvement in knee pain after dextrose injection, superior to that of synovial-fluid aspiration alone, and consistent with the speed and magnitude of analgesia reported upon injection of D5W into the caudal epidural space in patients with chronic low back pain [24]. Synovia- fluid SP levels more than doubled (112%, *p* = 0.028) at 1 week in the dextrose recipients. The between-group difference in SP elevation approached significance (*p* = 0.07), favoring the dextrose injection. The open-label study portion confirmed a sustained analgesic effect in the final follow-up (9 months), and a substantial (65%; *p* < 0.001) reduction in NPY levels from the baseline to 3 months. The short-term elevation of SP is of interest, as SP elevation, outside of the spinal cord, is associated with analgesia [25]. The medium-term elevation of NPY is also noteworthy, as NPY synovial-fluid levels rise commensurate with pain levels in knee osteoarthritis [20], and we demonstrated a substantial drop in both pain and NPY levels in these grade IV osteoarthritic pain patients with moderate-to-severe pain and functional impairment. 

Synovial aspiration also appears to be analgesic, although significantly less so than aspiration followed by dextrose injection. A potential benefit of synovial aspiration on analgesia in KOA is consistent with a randomized trial report of post-synovial-fluid aspiration analgesia lasting a week or longer [26].

It is of interest that the magnitude of analgesia measured 20 min after dextrose injection approximated the magnitude of analgesia observed at 9 months and was similar to the magnitude of improvement in pain reported in previous randomized trials using intra-articular only protocols [4,7] or intra-articular plus extra-articular protocols [6,8,9]. 

Although SP is often described in the literature to promote nociception [27], the promotion of nociception by SP may be confined to the spinal cord. Substance P has been identified as a primary neurotransmitter in antinociception induced by key descending influences of the lateral hypothalamus and periaqueductal gray [28]. Supraspinal SP injections reduced hyperalgesia and allodynia in a rat inflammatory-pain model [29]. Intramuscular SP injection induced antinociception in an acid-induced pain model [30]. The systemic administration of SP (intravenous) was antinociceptive in a neuropathic pain model [31]. A focus of the research into SP effects is on its activation of opioid receptors via NK1 receptor binding [18]. SP is being considered as a potential drug candidate in the treatment of neuropathic pain [31]. Thus, an increase in SP may represent a neurocytokine change consistent with analgesia. However, this was an isolated finding, without accompanying changes in CGRP or NPY. 

Multiple mechanisms for prompt dextrose-related analgesia are likely. Kim et al. proposed a potential “energy supplement” benefit of using dextrose in trigger-injection solutions [32]. John Lyftogt is identified as the proponent of the “energy hypothesis” related to chronic neuropathic pain [33]. In chronic pain states, peripheral C fibers and some A fibers manifest increased firing rates [34]. Recovery after firing requires ATP to power the ion pump for adequate repolarization, and glucose metabolism is the primary source of ATP production [35]. The energy hypothesis proposes that relative hypoglycemia is present in the sensory nerves, resulting in limited ATP availability and a failure of the ion pump to fully repolarize nociceptive nerve fibers to their normal -70 millivolts. Without adequate repolarization, the transmembrane potential of the nerve fiber sits too close to the firing threshold, resulting in an increased firing rate with stimulation or at rest [36]. MacIver reported that the firing rate of retinal C fibers rose markedly (>650%) within 20 min of the removal of dextrose from their in vitro nutritive solution, returning to baseline within 20 min of replacing dextrose in the solution [37]. 

At three months, we found a significant decrease in synovial-fluid concentrations of NPY. Given that Wang et al. reported higher NPY concentrations in knees with osteoarthritis, and progressively higher NPY concentrations as pain severity increased [20], the decrease in synovial NPY concentrations we observed suggests a mechanistic role of dextrose. However, studies on the role of NPY have indicated either anti- or pro-nociceptive actions [38], depending on differential receptor activation (NPY-1 versus NPY-2 receptors) [39], which we did not measure. The lack of information regarding NPY receptor concentrations, the fact that changes in NPY at 3 months were isolated (without changes in SP or CGRP), and the lack of data beyond the 3-month period point to the need for further research beyond the scope and power of this pilot study. 

We previously demonstrated a limited chondrogenic effect using interval arthroscopy, biopsy, and immunohistology in a small sample of grade IV KOA participants [40] treated by intra-articular injection with the same dextrose concentration and frequency of injection used in the present study. Our most prominent finding here in the non-neurogenic cytokines was an increase in IGF-1. Although IGF-1 stimulates the proliferation of chondrocytes in vitro, with chondroinductive actions equally potent to TGF-β [41], its increase in the current study was accompanied by a near-significant increase in a catabolic cytokine (IL-6). The non-neurocytokines we measured overlap only partially with Pan et al. [17], who measured changes in synovial-fluid cytokine concentrations in seven knees at 10 weeks, two weeks after completing five biweekly intra-articular injections of 25% dextrose, and in knees with less advanced KOA (Kellgren–Lawrence grade II-III). They reported significantly increased concentrations of matrix metalloproteinase 2, TIMP-1, epidermal growth factor, chemokine ligand 9, interleukin 10, and interleukin 22, only two of which would be considered either anticatabolic (TIMP-1) or anabolic (epidermal growth factor). We observed no significant changes in TIMP-1 in our 20 knees, but similarly found a combination of changes in catabolic and anabolic cytokines. 

A neuroprotective effect of brief glucose elevation in the nerve cells was suggested by an in vitro study by Wu et al. [42]. Human SH-SY5Y neuroblastoma cells, a commonly studied neuronal cell type, were exposed to tumor necrosis factor-alpha (TNF-α) in the presence or absence of glucose. Exposure to elevated glucose concentrations restored normal nerve cell metabolism, and reduced the production of inflammatory cytokines interleukin-6 and interleukin-1β, as well as cyclooxygenase-2 and nuclear factor kappa B, effectively blocking the unfavorable effects of TNF-α.

The primary limitations of our study were its small size, limited array of cytokines, and the non-blinding of the injector and assessor. Although the sample size was small, the effect size was large enough to detect some differences between groups. Expense constraints limited the frequency of ELISA measurements to three points in time, including the baseline. Given the extreme inflation in Argentina, associated with a concern among the general population about losing jobs due to taking unnecessary time off work, we were concerned about the loss of aspirate data at 6 and 12 months. Therefore, we elected to aspirate for ELISA analysis at baseline, one week, and three months. The strengths include a randomized design with a masked control for the initial phase, longer-term serial evaluation of the clinical outcomes, and the assessment of neurocytokine concentration as an outcome for the first time. 

## 5. Conclusions

In this pilot study, the intra-articular injection of dextrose in grade IV KOA resulted in prompt analgesia, a near-significant increase in synovial SP concentrations at one week, long-term analgesia at nine months, and a significant decrease in synovial NPY concentration at three months. The importance of these potentially favorable neurocytokine changes requires confirmation and clarification in larger studies, potentially with the inclusion of key neurocytokine receptors. At this time, the mechanisms of dextrose analgesia remain unclear, and they are likely multifactorial. 

## Figures and Tables

**Table 1:** Baseline Demographic Comparison Between Groups.

Measures	Dextrose 1st (n = 10)	Aspiration 1st (n = 10)	*p*
Age (SD)	65 (7)	71 (10)	0.13
BMI (SD)	31 (3)	30 (3)	0.30
0–10NRSPain (SD)	7.5 (1.3)	8.6 (1.4)	0.09
WOMAC (SD)	55 (9)	54 (25)	0.92

**Table 2:** Changes in 0–10 NRS scores.

Measures and Comparisons	Dextrose 1st (n = 10)	Aspiration 1st (n = 10)	Combined (n = 20)
NRS 0 ME (IQR)	7.5 (3.0)	8.5 (2.0)	8.0 (2.0)
NRS 20 min ME (IQR)	4.0 (4.0)	6.0 (4.0)	
NRS 0–20 min within group (raw score) ME (IQR); *p*	4.0 (3.5); <0.005	2.0 (5.3); 0.011	
NRS 0–20 min between groups (raw score) OR; *p*	OR = 9.03 ^1^; 0.05 ^1^		
NRS 1wk ME (IQR)	5.0 (4.0)	6.5 (3.0)	
NRS 0–1wk within group ME (IQR); *p*	2.0 (2.0); 0.005	1.0 (3.25); 0.041	
NRS 0–1wk between groups OR; *p*	OR = 4.48 ^1^; 0.14 ^1^		
NRS 3 months ME (IQR)	4.0 (5.0)	7.0 (7.0)	5.0 (6.0)
NRS 0–3 months within group ME (IQR); *p*	2.5 (5.5); 0.005	2.5 (5.3); 0.018	2.5 (4.5); <0.001
NRS 0–3 months between groups OR; *p*	OR = 2.36 ^1^; 0.37 ^1^		
NRS 6 months ME (IQR)	2.5 (5.0)	6.0 (4.0)	4.5 (5.0)
NRS 0–6 months within group ME (IQR); *p*	5.5 (4.8); 0.008	2.5 (3.5); 0.008	3.0 (4.0); <0.001
NRS 0–6 months between groups OR; *p*	OR = 4.06 ^1^; 0.19 ^1^		
NRS 9 months ME (IQR)	3.0 (6.0)	5.5 (5.0)	4.0 (5.0)
NRS 0–9 months within group ME (IQR); *p*	4.5 (3.0); 0.007	3.0 ± 2.25; 0.005	3.5 (3.5); <0.001
NRS 0–9 months between groups OR; *p*	OR = 2.03 ^1^; 0.44 ^1^		

^1^ These values represent the difference in scores between groups adjusted by the inclusion of NRS 0 as a covariate in proportional odds models. Odds ratio and *p*-value are presented.

**Table 3:** Time 0-week and 1-week cytokine concentrations in picograms/mL, adjusted for total protein concentration.

Cytokine	Time	Parameters	Dextrose 1st, (n = 10)	Aspiration 1st (n = 10)	Between Groups
SP	0	Mean (SD)	51 (50)	40 (29)	
	1 Wk	Mean (SD)	108 (124)	56 (82)	
	0 to 1 Wk	MD ^1^ (SE); *p*	+57 (28); *p* = 0.028 ^2^	+16 (22); *p* = 0.58	41 (35); *p* = 0.07 ^2^
CGRP	0	Mean (SD)	2.0 (0.9)	81 (99)	
	1 Wk	Mean (SD)	4.1 (3.5)	94 (77)	
	0 to 1 Wk	MD (SE); *p*	+2.1 (1.0); *p* = 0.14	+13 (34); *p* = 0.29	10.9 (34); *p* = 0.36
NPY	0	Mean (SD)	8.3 (5.2)	6.6 (8.0)	
	1 Wk	Mean (SD)	8.2 (3.7)	4.5 (4.7)	
	0 to 1 Wk	MD (SE); *p*	−0.1 (1.4); *p* = 0.80	−2.1 (2.9); *p* = 0.45	2.0 (2.3); *p* = 0.71
MMP-3	0	Mean (SD)	652 (694)	576 (609)	
	1 Wk	Mean (SD)	589 (345)	411 (339)	
	0 to 1 Wk	MD (SE); *p*	−63 (214); *p* = 0.24	−165 (131); *p* = 0.20	102 (251); *p* = 0.08 ^2^
TIMP-1	0	Mean (SD)	171 (122)	152 (60)	
	1 Wk	Mean (SD)	191 (79)	177 (95)	
	0 to 1 Wk	MD (SE); *p*	+20 (42); *p* = 0.29	+25 (34); *p* = 0.58	5 (54): *p* = 0.55
IL-6	0	Mean (SD)	20 (19)	21 (24)	
	1 Wk	Mean (SD)	26 (21)	34 (51)	
	0 to 1 Wk	MD (SE); *p*	+5.9 (7.5); *p* = 0.88	+13 (10); *p* = 0.28	6.8 (12.5); *p* = 0.33
IGF	0	Mean (SD)	5.4 (3.1)	5.3 (4.0)	
	1 Wk	Mean (SD)	5.0 (3.0)	5.5 (3.0)	
	0 to 1 Wk	MD (SE); *p*	−0.4 (0.8); *p* = 0.96	+0.2 (0.8): *p* = 0.80	0.6 (1.2); *p* = 0.71
TGFβ	0	Mean (SD)	91 (67)	135 (102)	
	1 Wk	Mean (SD)	105 (48)	140 (73)	
	0 to 1 Wk	MD (SE); *p*	+14 (22); *p* = 0.58	+5 (37); *p* = 0.96	9 (44); *p* = 0.94

^1^ MD = mean difference; ^2^ A Bonferroni-corrected alpha value of 0.006 was utilized to determine whether statistical significance was achieved.

**Table 4:** Cytokine concentrations in picograms/mL, adjusted for total protein concentration, and within group *p*-value for change from 0 to 3 months.

Cytokine ^1^	Time	Parameters	Glucose n = 20
SP	0	Mean (SD)	46 (40)
	3 Mo	Mean (SD)	39 (46)
	0 to 3 Mo	MD ^1^ (SE); *p*	−7 (13); *p* = 0.41
CGRP	0	Mean (SD)	42 (79)
	3 Mo	Mean (SD)	65 (121)
	0 to 3 Mo	MD (SE); *p*	+23 (27); *p* = 0.30
NPY	0	Mean (SD)	7.5 (6.6)
	3 Mo	Mean (SD)	2.6 (4.2)
	0 to 3 Mo	MD (SE); *p*	−4.9 (5.6); *p* < 0.0012
MMP-3	0	Mean (SD)	614 (637)
	3 Mo	Mean (SD	395 (211)
	0 to 3 Mo	MD (SE); *p*	−219 (129); *p* = 0.15
TIMP-1	0	Mean (SD)	161 (94)
	3 Mo	Mean (SD)	178 (63)
	0 to 3 Mo	MD (SE); *p*	+17 (28); *p* = 0.19
IL-6	0	Mean (SD)	21 (21)
	3 Mo	Mean (SD)	34 (32)
	0 to 3 Mo	MD (SE); *p*	+14 (7); *p* = 0.033 ^2^
IGF	0	Mean (SD)	5.4 (3.5)
	3 Mo	Mean (SD)	8.4 (4.7)
	0 to 3 Mo	MD (SE); *p*	+3.0 (0.9); *p* = 0.003 ^2^
TGFβ	0	Mean (SD)	113 (87)
	3 Mo	Mean (SD)	98 (68)
	0 to 3 Mo	MD (SE); *p*	−15 (22); *p* = 0.60

^1^ MD = mean difference; ^2^ A Bonferroni-corrected alpha value of 0.006 was utilized to determine whether statistical significance was achieved.

**Table 5:** Changes in the WOMAC scores.

Measures and Comparisons	Dextrose 1st (*n* = 10)	Aspiration 1st (*n* = 10)	Combined (*n* = 20)
WOMAC 0 months ± SD	55 ± 9	54 ± 25	54 ± 18
WOMAC 3 months ± SD	33 ± 14	46 ± 27	40 ± 22
WOMAC 0–3 months within group MD ± SE; *p*	22 ± 6; 0.004	8 ± 4; 0.09	14 ± 3.7; 0.001
WOMAC 0–3 months between groups MD ± SE; *p*	17 ± 7 ^1^; 0.031 ^1^		
WOMAC 6 months ± SD	23 ± 15	35 ± 25	29 ± 21
WOMAC 0–6 months within group MD ± SE; *p*	31 ± 4; <0.001	18 ± 4; 0.001	25 ± 3.1; <0.001
WOMAC 0–6 months between groups MD ± SE; *p*	11 ± 6 ^1^; 0.09 ^1^		

^1^ These values represent the difference in scores between groups adjusted by the inclusion of NRS 0 as a covariate in general linear model regression. Marginal mean difference between group and *p*-value are presented.

## Data Availability

The primary database for this study is available by request to the corresponding author.
